# Effect of an Oxadiazoline and a Lignan on Mycolic Acid Biosynthesis and Ultrastructural Changes of *Mycobacterium tuberculosis*

**DOI:** 10.1155/2011/986409

**Published:** 2011-04-11

**Authors:** Eduard Baquero, Wiston Quiñones, Wellman Ribon, Maria Leonor Caldas, Ladys Sarmiento, Fernando Echeverri

**Affiliations:** ^1^Grupo de Química Orgánica de Productos Naturales, Instituto de Química, Universidad de Antioquia, Carrera 53 No. 61-30, Laboratorio 234, Medellín 50010, Colombia; ^2^Grupo Micobacterias, Instituto Nacional de Salud, Bogotá 11001, D.C., Colombia; ^3^Grupo de Inmunología y Epidemiología Molecular, Universidad Industrial de Santander, Bucaramanga 68001, Colombia; ^4^Grupo Microscopia y Análisis de Imágenes, Instituto Nacional de Salud, Bogotá 11001, D.C., Colombia

## Abstract

Tuberculosis (TB) is an important disease that causes thousands of deaths around the world. Resistance against antitubercular available drugs has been reported; so, research on new effective antimycobacterial molecules is needed. Antimycobacterial activity of three lignans and two synthetic hydrazones was assessed against *Mycobacterium tuberculosis* H37Rv by antimycobacterial microdilution assay (TEMA). An oxadiazoline (AC451) and a lignan (ethoxycubebin) were the most active compounds (MIC 6.09 and 62.4 *μ*M, resp.). Several changes in mycolic acid profile of treated bacteria were detected with both compounds by mass spectrometry analysis. Additionally, the level of reduction of mycolic acids in ethoxycubebin treatment was correlated to disruption in bacterial morphology.

## 1. Introduction

Approximately 33% of the human population is infected with *Mycobacterium tuberculosis, *and every day 4,200 people die of tuberculosis around the world. Several facts have been considered as main factors for pandemic progress [1]. Antituberculosis drugs were discovered more than 40 years ago, [2, 3] resistant strains have emerged due to lengthy treatments, and latent and persistent infections have spread all over the world as population migration has increased. In addition and as it has been described recently, “HIV/AIDS continues to fuel tuberculosis epidemic, especially in Africa” [4]. Under these circumstances, there is an urgent need to search for new antimycobacterial substances to be used as templates for drug development so that the current situation of the disease can be tackled. Moreover, studies on the mechanism of action of new molecules can be useful to design more effective antimycobacterial compounds.****

Species of *Mycobacterium *and related genus synthesize mycolic acids (MAs), which are important lipid components of the cell wall (CW), are related to the protection of *M. tuberculosis *against dehydration, chemical injury, host immune system, and entry of hydrophilic antibiotics [5]. Therefore, MAs metabolism is an important target for *Mycobacterium *growth inhibition, for example, isoniazid is a first line antitubercular drug that inhibits MAs biosynthesis [6].

In this paper, we report the antimycobacterial activity of three lignans and two oxadiazoline derivatives against *M. tuberculosis *H37Rv analyzed by mass spectrometry and transmission electronic microscopy to assess the effect of the most active molecules on MAs biosynthesis and the ultrastructural changes of this pathogen.

## 2. Materials and Methods

### 2.1. Plant Material and Compounds

Leaves of *Virola flexuosa* were collected in Puerto Berrio (Antioquia-Colombia) in February 2007; a voucher was deposited at herbarium of Universidad de Antioquia (HUA 8967). 

### 2.2. Extract Preparation and Fractionation

Dried and ground plant material (1.0 kg) was extracted by percolation using hexane and ethanol (EtOH). Extracts were concentrated under vacuum, yielding crude extracts (CE) which were fractionated on a Sephadex LH-20 (Sigma) column using hexane-CH_2_Cl_2_-MeOH (2 : 1 : 1, v/v) to obtain several fractions. Subsequently, fractions were analyzed by ^1^H and ^13^C NMR spectroscopy to detect lignans, and finally lignans **1–3 **(70, 40 and 20 mg), respectively, were obtained by column chromatography procedures using silica gel 60 H (Merck) and hexane-EtOAc gradients.

**Synthesis of Compound 4**

1.2 mL acetic anhydride was added to pyridoxal isonicotinoyl hydrazone (35 mg, 0.122 mmol); the solution was left to stir at 105°C for 2 h and then worked up (extraction with CH_2_Cl_2_). Solvent removal gave a solid material, and column chromatography on silica gel (hexanes-EtOAc, 3 : 2) provided compound **4 **after chromatographic separation (12.14 mg, 0.037 mmol, 30.3%).

**Synthesis of Compound 5**

1.5 mL acetic anhydride was added to hydroxycinnamaldehíde benzhydrazone (50 mg, 0.198 mmol); the solution was left to stir at 151°C for 3 h and then worked up (extraction with CH_2_Cl_2_). Solvent removal gave a solid material, and column chromatography on silica gel (hexanes-EtOAc, 97 : 3) provided compound 5 after chromatographic separation (14.08 mg (0.048 mmol, 24%).

^1^H and ^13^C NMR spectra were recorded on Bruker AMX 300 spectrometer in CDCl3 (Compounds **1**, **2**, and **3**) or DMSO d6 (Compounds **4 **and **5**).

**Compound 4: *^1^H-NMR:***

H-5, *δ* 7.22 (*s, *1H); H-10, *δ* 8.29, (*s*, 1H); H-13, *δ* 7.59, (*d,* *J* = 5.4 Hz, 2H); H-14, *δ* 8.58, (*d*, 5.4 Hz, 2H); H-16, *δ* 2.07 (*s, *3H); H-17, *δ* 5.13, (*d, *12.9 Hz, 1H); H-17, 5.31, (*d, *12.9 Hz, 1H); 19-CH3, *δ* 1.85, (*s, *3H). *^13^C-NMR:* C-5, *δ* 89.16; C-2, *δ* 168.94; C-6, *δ* 130.79; C-7, *δ*155.57; C-8, *δ* 145.94; C-10, *δ* 149.21; C-11, *δ* 135.87; C-12, *δ* 132.97; C-13, *δ* 121.46; C-14, *δ* 152.12; C-16, *δ*19.27; 18-C=O, *δ* 171.21; 19-CH3, *δ*19.99.

**Compound 5: *^1^H-NMR:***

H-6a, *δ* 2.20 (*m*, 1H), 17-CH3, *δ* 2.22 (*s*, 3H); H6b, *δ* 2.35 (*m*, 1H); H-7, *δ* 2.72 (*m*, 2H); H-5, *δ* 6.25 (*d*, *J* = 2.7 and 5.9 Hz, 1H); H-9 and H-10, *δ* 7.15 (*m*, 4H); H- 13 and H-14, *δ* 7.40 (*m*, 4H); H-11 and H-15, *δ* 7.75 (*m*, 2H). *^13^C-NMR:* 17-CH3, *δ* 20.10; C-6, 29,59; C-7, *δ* 35,11, C-5, *δ* 92.74; C-12, *δ* 125,08; C-11 and C-15, *δ* 126,53; C-13 and C-14, *δ* 127,33; C-9 and C-10, *δ* 128.84 and *δ* 128.88; C-8, *δ* 140,85; C-2, *δ* 156,51; 18-C=O, *δ* 171.21.

### 2.3. Organism and Growth Conditions

*Mycobacterium tuberculosis *H37Rv (ATCC 27294) was first grown on Lowestein Jensen (LJ) medium for 15 days at 37°C. Bacterial suspension from LJ cultures were grown in Middlebrock 7H9 broth (Becton Dickinson) supplemented with 0.2% (by vol.) glycerol (Sigma), 10% (by vol.) OADC (oleic acid, albumin, dextrose, catalase; Difco), and 0.05% (by vol.) Tween 80 (Sigma). Cultures were incubated in 200 mL flask on a rotatory shaker at 100 rpm and 37°C for 12 d.

### 2.4. Antimycobacterial Activity

Antimycobacterial susceptibility was performed against *M. tuberculosis *H37Rv by 3-(4,5-dimethylthiazol-2-yl)-2,5-diphenyltetrazolium bromide (MTT) microdilution method (TEMA assay) as described by Porras et al. [7]. Minimum inhibitory concentration (MIC_90_) was defined as the lowest concentration that inhibits 90% of bacterial growth. Three different experiments were conducted in different days, and each compound was assessed in triplicate.

Stock solution of the compounds was dissolved in DMSO, stored at 4°C until use, and the final concentration of DMSO in experiments was less than or equal to 1%.

### 2.5. Mycolic Acid Analysis

*M. tuberculosis *H37Rv cultures (50 mL, 1 : 20 dilution of bacterial suspension adjusted 1 McFarland) were grown with or without compounds **3 **and **4 **at MIC, for 24, 48, and 72 h period. Bacterial cultures were centrifuged for 5 min at 4°C and 1048 g, and pellets were washed 5 times with cacodylate buffer (CB, 0.1 mol l-1; pH 7.2). Extraction and diazomethane derivation of mycolic acids were performed as described [8]. Methyl esters mycolic extracts (MEMEs) were analyzed by mass spectrometry (MS). MEMEs were resuspended in CHCl_3_:ACN (1 : 1, by vol.) and injected by means of pneumatically assisted pump in a mass spectrometer APCI-IT (Agilent series 6300). Mass spectra were acquired in negative mode and scan from 200 to 2000 m/z. Three different injections of each extract were carried out and the resulting data normalized and compared to data previously reported in the literature. 

### 2.6. Transmission Electron Microscopy (TEM)

Bacterial cultures were treated, centrifuged, and washed as mentioned above in MS analysis. Pellets were fixed with 2.5% Glutaraldehyde, 1% Ruthenium Red and 100 mM lysine in cacodylate buffer for 2 h at 4°C. After being washed with the same buffer, they were postfixed with 1% osmium tetraoxide and 0.05% Ruthenium Red for 2 h at 4°C. The cells were dehydrated in a series of ethanol, embedded in mixture of spur resin and propylene oxide at different proportions (2 : 1 and 1 : 1, by vol) and Spurr resin. Finally, they were polymerizated for 2 d at 68°C. Thin sections were stained with toluidine blue and observed under light microscopy. Ultrathin sections (60–90 nm) were stained with uranyl acetate and lead citrate. Finally, they were analyzed and photographed in Zeiss EM 109 electron microscope.

### 2.7. Cytotoxicity Assay

Cytotoxicity was determined according to MTT protocol assay described by [9]. The cell lines used were VERO and MDBK. The 50% inhibitory concentrations (IC_50_) of the assayed compounds were calculated by logarithmic regression analysis. Compounds were evaluated in triplicate.

## 3. Results and Discussion

**Figure 1:**
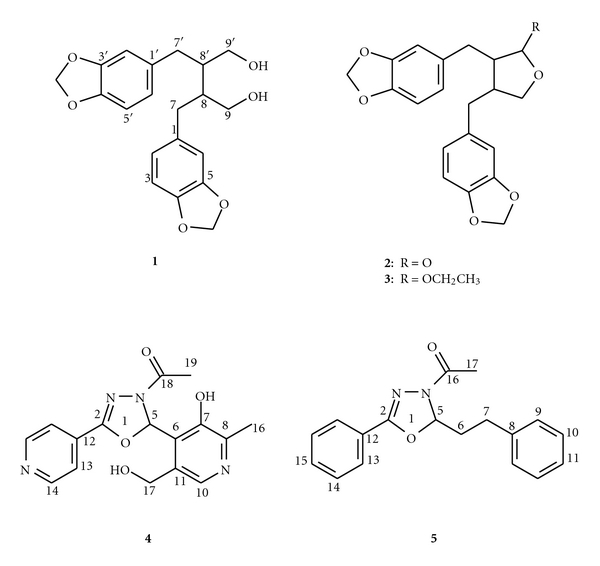
Lignans from *V. flexuosa *(**1, 2, **and **3**) and synthetic oxadiazolines (**4 **and **5**).

Lignans dihydrocubebin (**1**), hinokinin (**2**), and ethoxycubebin (**3**) (Figure 1) were previously isolated by us from *Virola flexuosa *(Myristicaceae) and reported for other plants, and their structural assignments were made by comparision of nmr spectra and physical properties [8, 10].****

Antimycobacterial and citotoxical activities of lignans and oxadiazolines are reported in Table 1. Compounds **3 **and **4 **showed the most potent inhibitory effect against *M. tuberculosis *H37Rv (MIC, 24 and 2 *μ*g mL^−1^, resp.). However, analogue compounds **2 **and **5 **were less active. MICs of reference drug were in accordance with the antibiotic susceptibility of the bacterial strain used. All compounds exhibited low cytotoxic effect compared to their antimycobacterial activity, with the exception of compound **5, **whose MIC_90_ was similar to IC_50_.

Due to the promising antimycobacterial activity and low citotoxicity of compounds **3 **and **4**, their effect on mycolic acid biosynthesis and ultrastructure was analyzed. Mass spectra of MEMEs were similar to spectroscopy data previously reported for MEMEs from *M. tuberculosis *H37Rv. However, there is a difference between the MS method employed by us with the one reported by other authors [11, 12]. This analysis allowed identification of alpha, keto, and methoxy mycolic acids in the samples analyzed (Table 2). Additionally, presence of [M^+^+Na] adducts of mycolic acids was confirmed by means of saturation of MEMEs with potassium salts as was reported [13]. 

**Figure 2:**
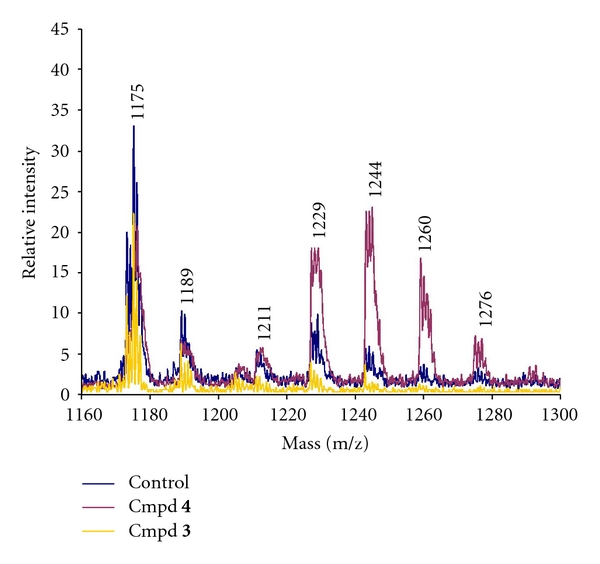
Mass spectrum of MEMEs from *M. tuberculosis *H37Rv cultures treated with ethoxycubebin (**3**) and AC451 (**4**) at 24 h.

MEMEs spectrum of bacteria treated with compound **4 **for 24 h showed an increase ranging from 180 to 480% in alpha and oxygenated mycolic acids with m/z ≥1225 (MA chain is equal to or greater than 82 carbons), and it also showed a reduction close to 50% in alpha mycolates with m/z ≤1189 (Figure 2). At the same time, compound **3 **caused a significant reduction in all mycolic acids. However, mycolic acid profiles at 48 and 72 h-treatment were similar to the control (data not shown).

**Figure 3:**
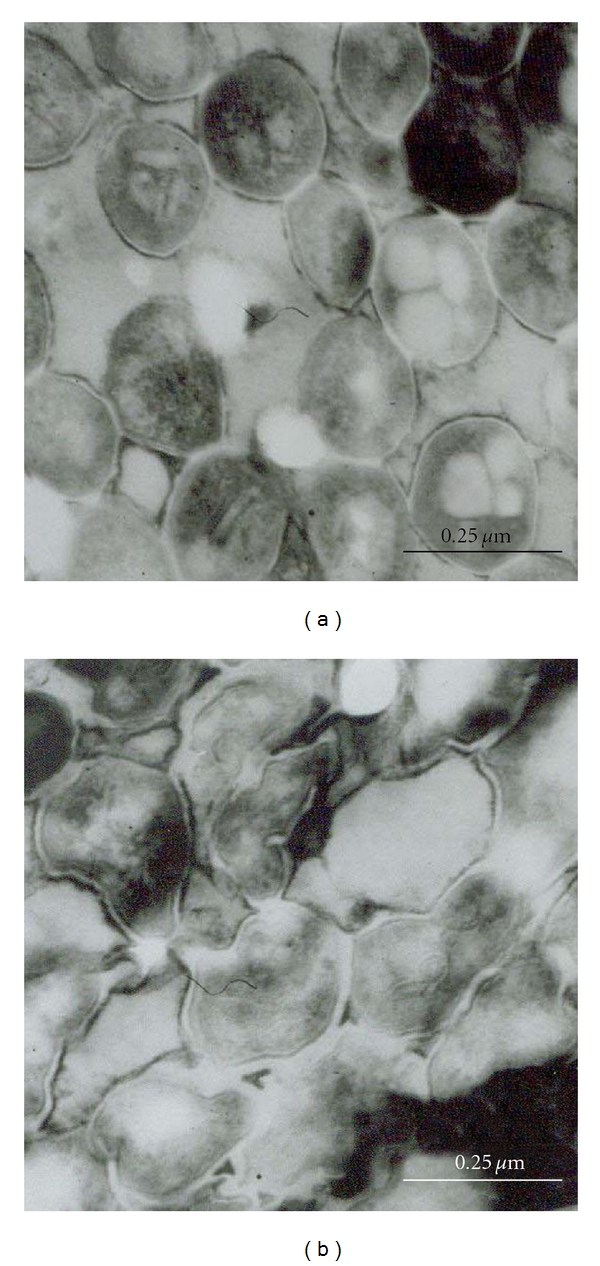
TEM images of *M. tuberculosis *H37Rv exposed to compound **3** at MIC for 24 h (b) and control (a). Plasma membrane (pm). Cell wall (cw). Mesosome (m). Bar: 13.5 mm.

Ultrastructural analysis of bacteria treated with compound **3 **exhibited aberrant morphology with irregular edges without disruption of the membrane and the cell wall from 24 h to 72 h of treatment (Figure 3, images at 72 h of treatment not shown). Nevertheless, the morphology of the bacteria treated with compound **4 **was not affected. Some control bacteria exhibited slight morphological alterations probably due to centrifugation process. 

However, the morphological alterations caused by the compounds were clearly different. In normal and abnormal cells, the cell wall (cw) was a moderately dense layer. The plasma membrane (pm) appeared to be composed of a double layer separated by a low density space. The inner layer here seems to be close together to bacterial cytoplasm, and the outer layer of the plasma membrane adhered closely to the cell wall. Additionally, TEM analysis of *M tuberculosis *bacteria showed similar lamellar structures to mesosome (m). In Figure 3(a), the plasma membrane is indicated with a black arrow head. Besides, some globules of various sizes in the cytoplasm, probably containing residual material from the culture, were observed. In Figure 3(b), in the region indicated by small arrows, the cytoplasm is hardly transformed and its shape is very irregular and disorganized.

In the present study, we assessed antimycobacterial activity of 3 lignans and 2 synthetic oxadiazolines against *M. tuberculosis *H37Rv in order to find new substances active on mycolic acids biosynthesis. As shown in Table 1, compound **3 **was the most active lignan tested. In this type of compound, the presence of an ethoxy substituent seems crucial for the activity, further evidenced by the difference in activity between compounds **2 **and **3**. Despite the absence of a furan ring in compound **1**, no significant difference was between the two compounds **1** and **2**, which suggests the importance of dibenzyl-methylene-dioxy butane moiety for the activity of these types of compounds. These lignans are promising since low levels of citotoxicity were observed. Additionally, antigenotoxic effect of hinokinin has been reported previously [14]. 

Oxadiazoline **4 **was the most active compound against *M. tuberculosis *and had the lowest cytotoxicity because MIC_90_ was 60 times lower than IC_50_. Other studies report oxadiazolines with a pyridine system similar to **4 **with promising antimycobacterial activity *in vitro *[15, 16]. Treatment of *M. tuberculosis *H37Rv with **4 **for 24 h exhibited alteration in MAs profiles, especially enhanced levels of keto and methoxy MAs. However, TEM analysis of bacteria treated with **4 **displayed normal morphology. It seems possible that over production of some MAs is a mechanism to overcome the effects caused by this compound on MAs metabolism, and can be related to the maintenance of normal bacterial morphology despite of treatment with compound **4. **There is evidence that some mutant and recombinant strains of *M. tuberculosis *producing higher proportions of some oxygenated MAs do not present aberrant morphology, as it happened in bacteria treated with **4 **[17]. 

On the other hand, reduced levels of all MAs observed in *M. tuberculosis *treated with lignan **3 **for 24 h can be related to an abnormal composition of the cell wall, and, therefore, caused aberrant bacterial morphology evidenced in the same treatment from 24 h to 72 h. Similar effects on mycolic acid biosynthesis and ultrastructural alterations have been reported in *M. tuberculosis *and *M. aurum *cultures treated with isoniazid, an antitubercular prodrug that inhibits mycolic acid biosynthesis [18]. To date, there are no reports on monofuranic lignans with effect against mycolic acid biosynthesis. 

As we mentioned above, alterations in MAs profiles were evidenced at 24 h of treatment with compounds **3 **and **4**. However, mass spectra of MEMEs at 48 and 72 h of both treatments showed MAs profiles similar to the control. This may mean that bacteria are restoring normal levels of MAs. Nevertheless, in the case of treatment with **3**, aberrant morphology was also evidenced at 72 h of treatment despite of MAs restoring profiles. This is due to a reversion of a cell wall deficient form, which is a complex process that in most of the cases cannot be accomplished even under ideal conditions [19]. 

Summarizing, we found lignan ethoxycubebin and oxadiazoline AC451 promising antimycobacterial compounds both having an effect on mycolic acid metabolism of *M. tuberculosis *H37Rv. These compounds can be useful templates for the development of more effective antimycobacterial derivatives that may be used in the future as antitubercular drugs. Also studies on the mechanism of action of these compounds can be useful to understand the metabolic processes related to MAs biosynthesis.

## 4. Conclusions

This paper reports antimycobacterial activity of two substances with effect on an important metabolic pathway of *M. tuberculosis*, that is, mycolic acid biosynthesis. These findings show that AC451 and ethoxycubebin are promising antimycobacterials affecting mycolic acid metabolism. These molecules are chemical templates to obtain more potent derivatives.

## Figures and Tables

**Table 1:** Antimycobacterial and cytotoxicity of lignans and oxadiazolines.

Compounds	MIC_90_ (*μ*M)	IC_50_ (*μ*M)	
		VERO	MDBK
**1**	159	≥698	≥698
**2**	169	≥706	≥706
**3**	62,4	203	≥651
**4**	6,09	—	367
**5**	81.1	—	9,34 × 10^−2^
*Isoniazid*	0,438	—	—
*Rifampin*	0,608	—	—

**Table 2:** Mass spectrometry data of MEMEs from *M. tuberculosis *H37Rv.

m/z peak	Type of mycolic acid*	Carbon number*
1175	Alpha	78
1189	Alpha	79
1211	—	—
1229	Alpha	82
1244	Keto	82
1260	Methoxy	83
1276	Keto	84

*Class and carbon number of mycolic acids in accordance with Laval et al. [12].
